# Hydraulic In Vitro Characterization of MR‐Conditional Blood Pumps

**DOI:** 10.1111/aor.70041

**Published:** 2025-11-17

**Authors:** Dominik T. Schulte, Marcel Renggli, Henning Richter, Francesca Del Chicca, Michael Hofmann, Martin O. Schmiady, Marianne Schmid Daners

**Affiliations:** ^1^ Institute for Dynamic Systems and Control ETH Zurich Zurich Switzerland; ^2^ Product Development Group Zurich ETH Zurich Zurich Switzerland; ^3^ Diagnostic Imaging Research Unit, Vetsuisse Faculty University of Zurich Zurich Switzerland; ^4^ Center for Cardiac Surgery, University Hospital Zurich University of Zurich Zurich Switzerland

**Keywords:** blood pump, cardiopulmonary bypass, extracorporeal membrane oxygenation, in vitro testing, MR‐conditional

## Abstract

**Background:**

Many cardiac surgical procedures use cardiopulmonary bypass (CPB), which in neonates requires the heart‐lung machine (HLM) to be positioned close to the patient due to their small circulating blood volume. The absence of an MR‐compatible blood pump to be used in CPB remains a key challenge for studying perioperative brain injury mechanisms in this high‐risk group. This study aims to take the first step toward MR‐conditional HLMs by developing hardware, verifying pump hydraulics, and ensuring MR compatibility.

**Methods:**

This study presents three MR‐conditional blood pump prototypes: a roller pump, a non‐occlusive roller pump, and a centrifugal pump. MR compatibility was assessed by monitoring for imaging interference during scanning. Hydraulic performance was evaluated with pressure—flow diagrams using a mock circulation test bench.

**Results:**

None of the prototypes interfered with MR imaging, and although SNR was reduced by −8.43% ± 7.96%, image quality remained sufficient for reliable assessment of relevant brain regions. Roller and non‐occlusive pumps maintained stable flow across pressure heads, with reductions of −4.46 ± 8.02 and −119.33 ± 18.22 mL/min, respectively. The centrifugal pump exhibited pressure‐dependent performance (slope −2.21 ± 0.45 mmHg/[L/min]). All pumps generated non‐pulsatile flow (SHE < 1000 erg/cm^3^).

**Conclusion:**

All three pumps meet basic MR‐conditionality and flow requirements, supporting their potential for use in MRI‐guided studies during neonatal cardiac surgery.

## Graphical abstract

Cardiac surgery in neonates requires close placement of cardiopulmonary bypass machines due to limited blood volume, complicating MR compatibility. This study introduces three MR‐conditional blood pump prototypes. None interfered with MRI imaging, though RF‐related sensor effects remain untested. Roller and non‐occlusive pumps delivered steady, pressure‐independent flow, while the centrifugal pump showed pressure‐dependent flow. All produced non‐pulsatile flow. The prototypes meet MR‐conditionality and flow requirements, offering potential for safe use in MRI‐guided neonatal cardiac surgery.

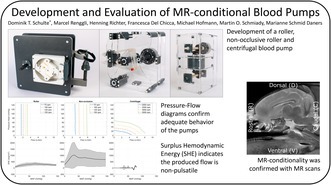

## 1 Introduction

Cardiopulmonary bypass (CPB) is used in cardiac surgery in neonates, for example, in the case of a single ventricle defect. By temporarily replacing the functions of the heart and lung with an external machine, the surgeon can operate on a non‐beating heart [1]. During such a procedure, the blood is continuously drained from the body from the superior or inferior vena cava and returned to the aorta, although alternative cannulation sites may be utilized if necessary [2]. The utilization of CPB is well established in adults and is used daily in approximately 2000 surgeries globally [3]; however, in recent years, multiple research groups have investigated the effects of employing heart‐lung machines (HLMs) on infants reporting conflicting results. Several studies have found preexisting brain injuries in 16%–32% of infants before surgery, with reported increases of 22%–36% immediately following cardiac surgery [4, 5, 6, 7]. On the contrary, a study by Bertholdt et al. [8] discovered a decrease of preoperative lesions a few days after surgery. Animal models have shown that CPB can reduce neurogenesis, impair cortical maturation [9], and alter white matter structure weeks after intervention [10, 11]. Whether this reflects slowed maturation or partial reversal of white matter growth remains unclear. To address this uncertainty, intraoperative magnetic resonance imaging (MRI) in experimental settings may provide unique insights into cerebral changes during CPB.

Currently MRI cannot be done during the surgery as the HLM cannot be used close to the MRI because of ferromagnetic components. Neonates have a very low blood volume of only around 275 mL [12, 13], with a proposed flow rate of 100 mL/kg bodyweight/min for neonates and 80 mL/kg bodyweight/min for pediatric cases according to the extracorporeal life support organization (ELSO) guidelines [14]. This requires the HLM tubing to be as short as possible to avoid large extracorporeal blood volumes, thus the pump and the oxygenator, needs to be close to the patient. One possible approach is to use long polycarbonate drive shafts to power the pump from outside the MR room [15]. Its use is limited because of its substantial spatial requirements. Chopski et al. [16] addressed this challenge by using a flexible drive cable in their investigation of mechanical cavopulmonary assistance, allowing pump operation with substantially reduced spatial requirements. Instead, our approach is to develop an HLM that can be used within the MRI without interfering with the imaging process and without endangering the patient.

An HLM can be operated with different pumps. A positive displacement or roller pump, compresses the tubing between two or more rollers and is mounted on a rotor against a fixed stator [17, 18]. A centrifugal pump on the other hand, is a cone‐ or fin‐type‐shaped rotating impeller which produces a pressure differential over the pump, causing a vortex, which results in flow [18]. Both pump types are clinically used. A third type is a non‐occlusive roller pump, where the tubing is stretched over three rollers to create a similar occlusion to a standard roller pump. As no fixed stator is used, the occlusion of the tube depends solely on the elongation of the specially manufactured flat tube [19, 20].

None of the concepts are MR‐conditional, however, the feasibility of an MR‐conditional HLM has previously been shown [21]. The use of an MR‐conditional HLM enables the investigation of changes in cerebral perfusion dynamics during cardiac surgery. The goal of this study is to take the first step toward the development of MR‐conditional heart–lung machines. To this end, we focus on the design and construction of the hardware, the verification of the hydraulic performance of the pumps, and the assessment of potential interactions between the pumps and the MR environment.

## 2 Materials and Methods

### 2.1 Prototypes

**FIGURE 1:**
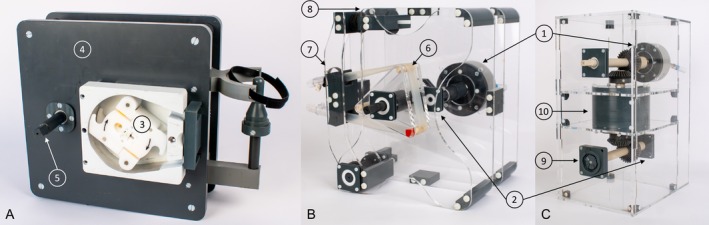
The three developed MR‐conditional pumps. The PM0450 air motor (1) and the ME 22 LD encoder (2) were used in all three pumps (cannot be seen in A). (A) Shows the roller pump with the SPQ 225 pump head (3), the PVC pump body (4), and the emergency crank (5). (B) Shows the non‐occlusive roller pump with its three polymer rods (6), and the tensioning arm (7) that can be adjusted with a threaded rod (8). (C) Shows the centrifugal pump with the mechanical transmission (9) to the DP2 pump head and the two‐stage planetary gears (10) [22]. [Color figure can be viewed at wileyonlinelibrary.com]

All three prototypes (Figure 1) were built with the same PM0450 air motor (PTM Mechatronics GmbH and Bibus AG, Fehraltorf, CH) for actuation (Figure 1, No. 1), the ME 22 LD encoder (PWB encoders GmbH, Eisenach, DE) to measure the rotor position and the rotational speed (Figure 1, No. 2), and the Tecno Basic Pre‐U piezo valve (Hoerbiger Holding AG, Zug, CH) to control the airflow of the motor, same as the prototype by Hofmann et al. [21].

#### 2.1.1 Roller Pump (RP)

Figure 1A: The prototype comprises the SPQ 225 (Möller Medical GmbH, Fulda, DE) roller pump head (Figure 1, No. 3), a pump body of milled polyvinyl chloride (PVC) (Figure 1, No. 4) and gears made from polyoxymethylene (POM) with a ratio of 3:1. Closed‐cell polyurethane foam (Sylodyn NC, Getzner Werkstoffe GmbH, Bürs, AT) was used to suspend the two rollers of the pump head. The air motor and the shielded encoder are mounted on the back of the pump body, and the pump head and the access shaft for the emergency crank on the front (Figure 1, No. 5). The pump can be operated with standard medical 1/4″ or 3/8″ tubing (HMT Medizintechnik GmbH, Maisach, DE).

#### 2.1.2 Non‐Occlusive Roller Pump (NRP)

Figure 1B: Inspired by the design of Montoya et al. [23], a prototype was constructed from milled PVC and acrylic plates. Standard 1/2″ silicone tubing (HMT Medizintechnik GmbH, Maisach, DE) is stretched over three polymer rods (Figure 1, No. 6). This tubing is mounted on the tensioning arm of the pump (Figure 1, No. 7); one side is fixed with two ceramic ball bearings, the other can be moved with a threaded polyamide rod and a hollow rubber spring (HIB 1842‐M‐350, Vibraplast, Aadorf, CH) (Figure 1, No. 8). This mechanism allows for the stretching force on the tubing to be dynamically adjusted.

#### 2.1.3 Centrifugal Pump (CP)

Figure 1C: A modified DP2 centrifugal pump (MEDOS Medizintechnik AG, Heilbronn, DE) is used as the pump head for this prototype. To ensure the MR‐conditionality of the pump head, the shaft was replaced with a polyetheretherketone (PEEK) shaft, and the ball bearings with ceramic bearings. Since the magnetic coupling cannot be used in the MRI, the back of the pump was opened, and a milled PVC piece replaced the metal transmission piece which was used for magnetic transmission of rotational movement. This transmission piece is a circular plate with saw teeth on top. This piece is mounted to the pump and the transmission such that the flat side of the teeth transmits the rotational motion while also allowing the pump head to be easily replaced (Figure 1, No. 9). The required rotational speed is achieved by employing a two‐stage planetary gear set with thermoplastic gears (Stagnoli TG s.r.l., Lonato, IT) having a combined ratio of ~1:40 in the center of the pump body (Figure 1, No. 10). Two sets of bevel gears were used, one before and one after the planetary gears, such that the emergency crank can be attached between the transmission and the motor.

### 2.2 MR Conditionality

To safely operate the pump inside the MR scanner, MR conditionality needs to be ensured. Even though the use of ferromagnetic components was avoided, some components still contain metals. The PM0450 motor contains multiple brass and aluminum components, the outer body of the SPQ 225 roller pump is milled from aluminum, and the encoder is encased in copper shielding and can itself be affected by radiofrequency interference. Those components could cause artifacts in the imaging process or experience interference due to the strong radiofrequency pulses of the MR scanner.

This interaction was evaluated by placing the roller pump inside the MR scanner and operating the pump with constant air supply. An ultrasonic flow probe (Sonoflow CO.55, Sonotec, Halle, DE) in an aluminum shielding with waveguides was placed inside the scanner as well to detect any interferences caused by the metallic casing. The interaction was only evaluated with the roller pump as it contained by far the most metallic components due to the SPQ 225's outer body.

A post‐mortem piglet head was positioned in the head coil (dStream Head Neck, 32ch, Philips AG Healthcare, Horgen, CH) of a 3 Tesla Philips Ingenia Elition X (Philips, Amsterdam, NL) MR scanner to simulate in vivo conditions, enabling the detection and assessment of small‐scale imaging artifacts relevant to the intended application. The MR‐conditional pump was placed at the entrance of the scanner bore, with the flow sensor positioned inside the bore near the estimated anatomical location of the heart. The motor's rotational axis was aligned with the scanner bore to minimize the induction of eddy currents in the rotating shaft. No tubing was inserted into the pump or connected to the post‐mortem piglet head, as the focus of this experiment was solely to detect interactions between the pump and the MR scanner.

Morphological images were acquired using a T2‐weighted turbo spin‐echo sequence (repetition time/echo time = 3000/80 ms; acquisition matrix = 184 × 151; slice thickness = 1.5 mm) in sagittal and transversal orientations. Additionally, diffusion tensor imaging (DTI) scans were acquired in dorsal orientation using the following parameters: repetition time/echo time = 1629.89/86.30 ms; acquisition matrix = 68 × 66; slice thickness = 2 mm; *b* = 1000 s/mm^2^; 32 diffusion‐encoding directions. The T2‐weighted sequences took 216, 343 and 336 s for sagittal, transversal and dorsal orientation, respectively, and 161 s per iteration for the DTI scans.

All images were reviewed by an MR specialist from the Vetsuisse Faculty, University of Zurich, to assess potential distortions that could interfere with analysis in future in vivo studies. The signal‐to‐noise ratio (SNR) was calculated as the mean signal intensity within a given brain region divided by the standard deviation of an equally sized region of interest (ROI) placed in the image background according to the single‐image method described by McCann et al. [24] The true SNR is recovered by multiplying the calculated value with the Rician distribution (0.655). Five regions were assessed: the basal ganglia, hippocampus, internal capsule, centrum semiovale, and medulla oblongata, all of which are known to be particularly sensitive to perfusion changes, such as those observed in perinatal asphyxia [25, 26, 27, 28, 29]. A non‐parametric Wilcoxon signed‐rank test was conducted to compare the mean SNR before and after inserting the pump into the MR scanner and determine if a statistically significant change has occurred. In addition, the rotational function of the pump was monitored using the encoder to determine whether the strong magnetic field affected pump operation or sensor readout.

### 2.3 Hydraulic Performance Evaluation

The hydraulic performance of all three prototypes was evaluated in a next step by their pressure‐flow characteristics. Therefore, the pressure head‐flow (HQ) diagrams were recorded on the hybrid mock circulation (HMC) test bench [30]. An HQ diagram shows the changes in the flow rate caused by changes in the pressure head, which describes the pressure difference between downstream and upstream of the pump.

**FIGURE 2:**
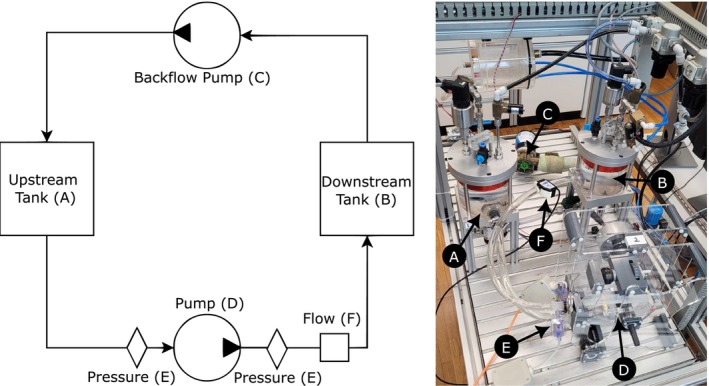
The central components of the hybrid mock circulation (HMC) test bench [30] are two water tanks that are pressurized to mimic varying pressures upstream (A) and downstream (B) of the pump. To ensure that both tanks have the same water level, a backflow pump (C) is controlled to feed the pumped water back from the downstream tank into the upstream tank, closing the loop. Our pump (D), here represented by the non‐occlusive roller pump, was connected by tubing connectors to both tanks. The generated pressure is measured directly before and after the pump with two piezoelectric pressure sensors (E) (TruWave, Edwards Lifesciences, Irvine CA, US). The blood flow by the pump is measured with an ultrasonic flow probe (F) (TS410/ME‐11PXL, Transonic Systems Inc., Ithaca NY, US). [Color figure can be viewed at wileyonlinelibrary.com]

A sketch of the hardware setup is shown in Figure 2. As mentioned before, the proposed flow rate for neonates and pediatric cases is 80–100 mL/kg bodyweight/min, which leads to a required flow range of only around 320–1200 mL/min, assuming an average weight of 15 kg after 3.5 years after birth [12]. This flow rate is much lower than the average flow rate of an adult of 4200 mL/min during CPB (calculated from an average CPB cardiac index of 2.34 L/min/m^2^ and an average body surface area of 1.79 m^2^) [31, 32]. Instead, the flow range fits to the lower range of the 0.5–4.0 L/min normally used in extracorporeal carbon dioxide removal applications (ECCO_2_R). This system is generally used only for CO_2_ removal and not to replace the heart's function, but since the system is operated at low flow points it satisfies the flow requirements for pediatric CPB which is why the reference pressures for our experiment are taken from ECCO_2_R application [33].

The HMC was set up such that the upstream tank at the pump inlet remained at a constant pressure of 0 mmHg while the downstream tank at the outlet increased the pressure by 5 mmHg every 15 s from 0 to 150 mmHg. The constant 0 mmHg in the upstream tank mimics a physiological central venous pressure [34]. The downstream tank mimics the arterial pressure in this setup. The selected pressure range was determined based on the operational requirements of a low‐flow ECCO_2_R system, where 150 mmHg is the lower pressure head target [33]. This process was repeated five times for every prototype at four different rotational speeds of 75, 100, 125, and 150 rpm for the roller pumps and 2000, 2500, 3000, and 3500 rpm for the centrifugal pump. The rotational speed of the pump was held constant by using the same PID controller as for the MR‐conditionality experiments to control the airflow through the piezo valve according to the measurement of the encoder.

The HQ diagram serves as a direct means to evaluate whether the pump can generate sufficient flow for the patient. The steepness of the HQ curve at the intended operating point was analyzed by calculating the two data points closest to a flow rate of 1000 mL/min for each speed.

The pulsatility of the developed pumps was quantified by calculating the surplus hemodynamic energy (SHE) (Equation 1), which describes the added energy into the blood by the pump during pulsatile flow generation:

(1) $$\mathrm{SHE}=1332*\left(\mathrm{EEP}-\mathrm{MAP}\right)$$

With energy equivalent pressure (EEP) (Equation 2) and mean arterial pressure (MAP),

(2) $$\begin{array}{c} \mathrm{EEP}=\frac{\int P*Q\;\mathrm{dt}}{\int Q\;\mathrm{dt}} \end{array}$$

With *P* being the arterial pressure and *Q* being the flow according to Shepard et al. [35] The MAP is calculated by integrating the pressure over the time interval utilizing the trapezoidal numerical integration method. The raw flow and pressure data acquired for the HQ diagrams were used for these calculations [34]. Exact SHE values are reported for the lowest standard pressure during CPB (40 mmHg) [36] and hypertension (100 mmHg) [37].

## 3 Results

### 3.1 MR Conditionality

**FIGURE 3:**
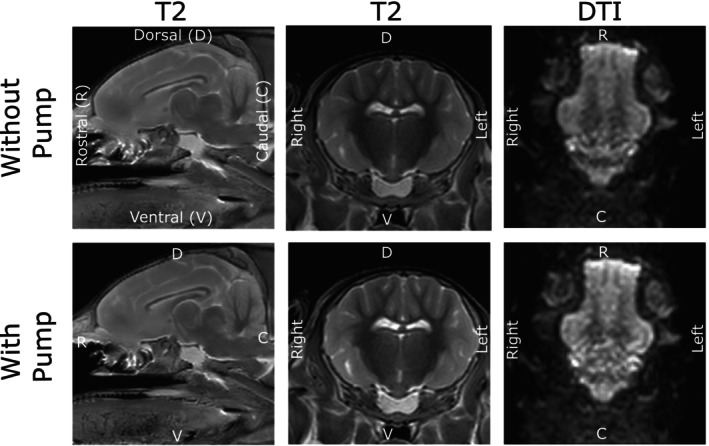
The figure shows the T2 morphological scans in sagittal and transversal view and DTI scans in dorsal view before and after the MR‐conditional roller pump was placed inside the bore of the MR scanner.

Figure 3 summarizes MRI scans of a post‐mortem pig head acquired using a 3 T Philips Ingenia scanner, both before and after placement of the pump inside the MR environment. The morphological T2‐weighted Turbo Spin Echo sequence provided sufficient resolution to assess potential tissue injury. Although the resolution of the diffusion tensor imaging (DTI) was lower, it was adequate for a preliminary evaluation of diffusion dynamics. No imaging artifacts were observed in the morphological or DTI scans across any brain region, and no signal disturbances were detected in the encoder output or pump rotational speed.

Analysis of the SNR measurements revealed an average decrease in SNR by −8.43% ± 7.96% over all measured regions. The exact results are displayed in Table 1. A non‐parametric Wilcoxon signed‐rank test was performed to compare values before and after the intervention in nine samples. The analysis revealed a significant decrease after the intervention (*V* = 2, *p* = 0.0059). The mean difference was −4.68 units (95% CI: −7.54 to −1.82), indicating that post‐intervention values were significantly lower than pre‐intervention values. This confirms that the intervention induced a measurable reduction in SNR.

**TABLE 1:** Signal‐to‐noise (SNR) ratio of different brain regions without (w/o) the pump and with (w) the pump placed at the entrance of the MR bore. Most brain regions differ in left and right areas. The change in SNR is given as a difference in % from the initial SNR value.

Region		SNR w/o pump	SNR w pump	Difference
Basal ganglia	Left	47.35	43.23	−8.71%
	Right	60.79	55.53	−8.65%
Hippocampus	Left	93.66	89.04	−4.94%
	Right	87.40	86.01	−1.59%
Internal capsule	Left	34.66	36.75	6.03%
	Right	43.67	35.28	−19.22%
Centrum semiovale	Left	51.39	42.06	−18.14%
	Right	50.10	41.68	−16.81%
Medulla oblongata	Midline	70.44	67.77	−3.80%
Average		59.94 ± 18.96	55.26 ± 19.69	−8.43% ± 7.96%

### 3.2 Hydraulic Performance Evaluation

**FIGURE 4:**
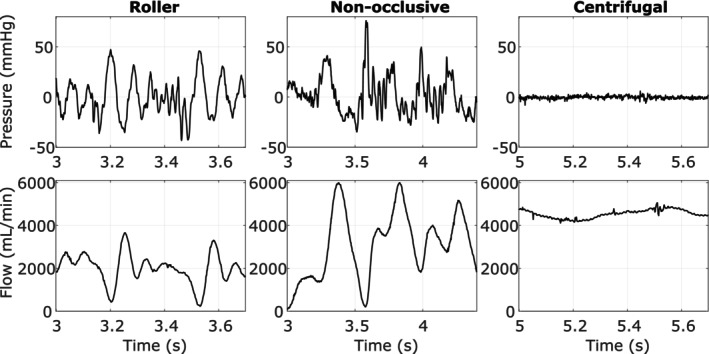
The measurements for both plots were taken at a pressure head of 0 mmHg. The top row shows exemplary and representative outlet pressure of the roller pump (RP), the non‐occlusive roller pump (NRP), and the centrifugal pump (CP) as measured on the mock circulation test bench. The bottom row shows the corresponding flow profiles. The *x*‐axis differs between the pumps since the times were chosen such that the shown curves best represent the characteristics of the pumps.

Analyzing the flow and pressure curves of the three prototypes at a pressure head of 0 mmHg shows fluctuating outlet pressure on both the roller and non‐occlusive roller pumps, between −43.31 to 47.20 mmHg and −34.83 to 76.01 mmHg respectively, whereas the centrifugal pump maintained a constant pressure of −0.41 ± 1.44 mmHg (Figure 4). Similarly, the roller pump and non‐occlusive roller pump produced oscillating flow patterns of 2001.60 ± 702.55 mL/min and 3102.70 ± 1545.80 mL/min respectively, whereas the centrifugal pump displayed a slower oscillating flow of 4540.10 ± 197.00 mL/min.

**FIGURE 5:**
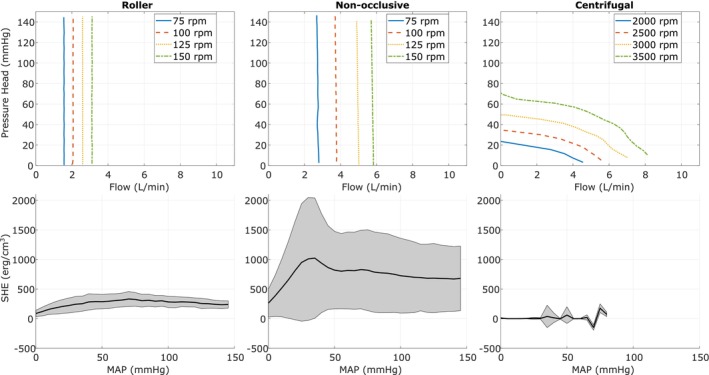
The top figure shows the flow characteristics of the roller pump, the non‐occlusive roller pump, and the centrifugal pump when increasing the pressure difference over the pump (pressure head) from 0 to 150 mmHg at four different speeds. The bottom figure shows the surplus hemodynamic energy (SHE) as an average (black line) with standard deviation (gray area) as mean arterial pressure (MAP) is increased. [Color figure can be viewed at wileyonlinelibrary.com]

The hydraulic performance experiments have shown that roller and non‐occlusive roller pumps can produce mostly constant flows independent of the pressure head. The flow rates and measured pressure heads of these two pumps are shown in Figure 5 for four different rotational speeds of 75, 100, 125, and 150 rpm at the first and last pressure setpoints of the respective experiment. The roller pump and the non‐occlusive roller pump produced steady flows ranging from 1566.1 ± 16.5 to 3113.9 ± 12.3 mL/min and 2798.2 ± 10.4 to 5827.8 ± 236.2 mL/min, respectively. For the centrifugal pump, Figure 5 shows the measured flow rate and pressure head for four rotational speeds of 2000, 2500, 3000, and 3500 rpm at the start of the experiment, when both tanks are set to equal pressure. Additionally, for each rotational speed, the approximate pressure head is given when the pump stops producing flow.

The steepness of the HQ curve of the centrifugal pump at around 1 L/min from 2000 to 3500 rpm in steps of 500 rpm was: −2.87 mmHg/(L/min), −2.12 mmHg/(L/min), −2.20 mmHg/(L/min), and −1.84 mmHg/(L/min).

Figure 5 bottom panels show the SHE for the three pumps as an average with standard deviation. The detailed measurements and their standard deviations are given in Table 2.

**TABLE 2:** The results from the HQ diagram and the surplus hemodynamic energy (SHE) calculations are summarized for each of the three developed pumps. For the roller and the non‐occlusive roller pump, the average flow rate is given for the two different pressures (i) and (ii) that correspond to the minimum and maximum measured head pressure for the respective four different rotational speeds. The flow and resulting pressure head with both tanks being set to the same pressure is given for the centrifugal pump for four different rotational speeds with no resistance corresponding to respective maximum flow. Additionally, the approximate pressure head when the pump produces zero flow is given. The SHE is given at two different mean arterial pressures (MAP) for all three pumps.

Roller			Non‐occlusive			Centrifugal			
**HQ‐Diagram**									
	**(i)**	**(ii)**		**(i)**	**(ii)**	**No Resistance**			**Zero flow**
**Pressure (mmHg)**	1.25 ± 0.55	145.17 ± 0.39	**Pressure (mmHg)**	0.54 ± 3.33	144.57 ± 3.46				
**Speed (rpm)**	**Flow (mL/min)**		**Speed (rpm)**	**Flow (mL/min)**		**Speed (rpm)**	**Pressure (mmHg)**	**Flow (mL/min)**	**Pressure (mmHg)**
75	1566.1 ± 16.5	1552.7 ± 28.6	75	2798.2 ± 10.4	2681.4 ± 36.3	2000 rpm	3.22 ± 0.43	4558.2 ± 85.47	~24 mmHg
100	2033.0 ± 64.3	2069.0 ± 10.7	100	3775.5 ± 97.3	3675.3 ± 103.1	2500 rpm	5.29 ± 0.49	5559.0 ± 458.17	~35 mmHg
125	2593.0 ± 6.3	2590.1 ± 10.0	125	5021.9 ± 93.3	4898.7 ± 67.5	3000 rpm	7.93 ± 0.47	7011.5 ± 33.54	~50 mmHg
150	3113.9 ± 12.3	3116.2 ± 8.9	150	5827.8 ± 236.2	5698.5 ± 252.9	3500 rpm	10.47 ± 0.31	8121.9 ± 61.12	~70 mmHg

Roller			Non‐occlusive			Centrifugal			
**Surplus Hemodynamic Energy**									
**MAP (mmHg)**	**SHE (erg/cm** ^**3**^ **)**		**MAP (mmHg)**		**SHE (erg/cm** ^**3**^ **)**	**MAP (mmHg)**		**SHE (erg/cm** ^**3**^ **)**	
40.12 ± 0.57	283.79 ± 136.07		40.06 ± 0.44		939.43 ± 837.81	40.02 ± 0.40		15.16 ± 80.46	
100.20 ± 0.58	282.65 ± 93.81		100.14 ± 0.46		725.69 ± 631.23	80.05 ± 0.31		72.79 ± 36.51	

## 4 Discussion

During open‐heart surgery the patient's cardiovascular system and oxygenation are supported by an HLM. To improve the understanding of brain perfusion dynamics during such surgeries, particularly in pediatric patients, MRI can be used to assess brain tissue integrity and blood flow non‐invasively. MRI was previously used to assess white matter brain injuries before and after surgery in infants [4, 5, 6, 7, 8]. To enable MRI while the patient is supported by an HLM, three prototypes were developed, namely a roller pump, a non‐occlusive roller pump, and a centrifugal pump, each satisfying the requirements of being MR‐conditional. Afterwards, these prototypes' hydraulic performance was analyzed on an HMC to assess the feasibility of such a pump for preclinical use.

### 4.1 MR Conditionality

Instead of using a simple phantom, a post‐mortem piglet head was chosen to reflect the intended in vivo application of the pump in piglet studies. The pump was not connected to the head and operated adjacent to the piglet at the borehole in a purely standalone mode to simulate normal operating conditions.

Morphological images are essential for detecting tissue injury [4, 5, 8], while DTI scans allow assessment of early tissue changes before structural damage occurs [25]. The roller pump did not introduce any imaging artifacts, confirming its MR compatibility.

Although our MR‐conditional setup produced a reduced signal‐to‐noise ratio (SNR), the resulting image quality remained sufficient for reliable ROI‐based analysis. Specific crucial anatomical brain structures, including the basal ganglia, hippocampus, and internal capsule, were consistently visualized with unchanged image quality and no visible signs of degradation. In practical settings, absolute SNR thresholds vary; nevertheless, reductions of approximately 10 dB, as reported by Shokrollahi et al. [38] in motor‐induced artifact studies, were still deemed acceptable for image assessment. We also observed notable variability in our SNR measurements, consistent with McCann et al. [24], who highlighted the inherent variability in SNR assessment methods across regions. Taken together, these findings suggest that while further optimization of SNR and reducing variability remain important aims for future MR‐conditional design, the current level of image quality supports the feasibility of in vivo MRI investigations into neonatal cerebral perfusion dynamics.

Given that the non‐occlusive roller and centrifugal pumps contain even fewer metallic components, these findings support the safe operation of all three pump types within the MR environment, even without testing the latter two prototypes. No disturbances were observed in the rotation of the roller pump or in the operation of the encoder, indicating that the pump can operate safely and uninhibited in close proximity of the MR scanner.

### 4.2 Hydraulic Performance Evaluation

Pressure fluctuations were detected at the outlet of the roller and the non‐occlusive roller pump. The roller pump produced a prominent pressure peak, followed by a decaying outlet pressure oscillation pattern, as expected of a positive displacement pump [39, 40]. The HQ plot for the roller pump showed a fixed flow rate, depending on the speed. By increasing the pressure head over the pump, the flow was only reduced by −4.46 ± 8.02 mL/min, which is a central characteristic of the positive displacement pump [41].

The non‐occlusive roller pump showed more extensive and more irregular changes in outlet pressure than the roller pump, which differs from previous findings [23]. The non‐occlusive roller pump had a higher reduction of flow (−119.33 ± 18.22 mL/min) than the roller pump, which was expected since the tubing was not squeezed against a rigid shell. The main difference to what Montoya et al. [23] found is that a change in sensitivity was not observed. In particular, Montoya and coworkers observed that with an increasing pressure head, the sensitivity initially remained low as with a roller pump during low pressure head and suddenly increased at a higher pressure head as is normally observed with a centrifugal pump. This lack of change in sensitivity is assumed to be mainly due to the maximum pressure head in our study being 150 mmHg, while in their work, this sudden reduction in flow only appeared at pressures above 150 mmHg.

The centrifugal pump displayed a HQ curve shape expected based on the working principle of the pump [18, 41, 42, 43]. Nonetheless, the steepness of the HQ curves could be used to characterize the pump further. A flatter curve has the advantage that the suction at the inlet is lower at low flows, such as in pediatric use [44]. An average gradient of −2.21 ± 0.45 mmHg/(L/min) was observed for the centrifugal pump at 1 L/min, which is close to −0.94 mmHg/(L/min) [45] and −3.78 mmHg/(L/min) [46]. This is a very low steepness, resulting in a high pressure sensitivity at low flows, which would be optimal for our proposed application.

The hydraulic flow experiments show that the flow rate of the roller and non‐occlusive roller pump was higher than the desired flow rate of 1200 mL/min for pediatric use [14]. This can be adjusted by changing the tubing diameter from 3/8″ for the roller pump to 1/4″ and from 1/2″ for the non‐occlusive roller pump to 1/4″. The centrifugal pump was highly sensitive to pressure changes around a flow of 1 L/min. To reach this flow at a pressure head higher than 100 mmHg, the speed of the pump would need to be increased further, which is not possible with this motor and planetary gears. Additionally, according to Tan et al. [47], the roller and non‐occlusive roller pumps are more suitable to generate pulsatile flow, although pulsatility was insufficient in our setting for all three pump types.

The debate about pulsatility in cardiac assist devices has been ongoing for decades without a consensus in the field [47, 48]. Two studies by Ündar et al. [49, 50] have shown that pulsatility is an important aspect in the direct comparison of different pumps. The surplus hemodynamic energy is calculated from the pressure of the tank to evaluate the pulsatility acting inside the pseudo patient's arteries. The calculated SHE was very low for all three pumps. The non‐occlusive roller pump had the highest calculated average energy out of all three pumps (939.43 ± 837.81 erg/cm^3^ at a mean arterial pressure of 40.06 ± 0.44 mmHg) but still generated much less than the 7726 ± 1757 erg/cm^3^ calculated for the Stockert SII mast‐mounted pulsatile roller pump, which was deemed “sufficiently pulsatile” by Ündar et al. [49] While the roller pump produced around 280 erg/cm^3^ of surplus energy, the centrifugal pump only produced around 60 erg/cm^3^. The result for the centrifugal pump falls within a range consistent with previous research, where in Wang et al.'s work [45], non‐pulsatile flow, as expected from a centrifugal pump, was suggested to produce 0 erg/cm^3^. One study by Ündar et al. [50] showed that the oxygenator and the tubing caused the biggest loss of energy in a complete surgical setup. Our setup was strongly simplified in comparison since our study aimed to only characterize the pumps alone. Adding the oxygenator and longer tubing to our pumps would therefore decrease the SHE further.

Roller and centrifugal pumps are generally considered to generate non‐pulsatile flow, which remains the predominant mode of perfusion in cardiac surgery [47]. However, even with a lack of strong evidence for beneficial effects, the 2019 EACTS/EACTA/EBCP guidelines on CPB in adult cardiac surgery recommend pulsatile perfusion, specifically in patients at high risk of complications relating to the lungs and the renal system [51]. In practice, pulsatility is typically achieved either by modulating pump speed or by integrating an external pulsator into the circuit [47]. An example for such a pulsator utilizes a piston pump after a roller pump to convert continuous flow into pulsatile flow [52]. Both approaches could also be applied to the prototypes presented here, as their design is independent of the specific blood pump used.

## 5 Limitations

The main limitation in this study is that the pump was not operated with an electrical system. Even though the large metal components did not induce any interference with the MR scanner, the electrical components could potentially be interfered with by the strong radiofrequency signals of the MR scanner. It is important to shield all the electrical components appropriately and verify their functionality in a next step, before the in vivo trials are conducted.

## 6 Conclusion

An MR‐conditional blood pump allows to use MRI as a diagnostic method for early recognition of changes in cerebral perfusion during cardiac surgery. Our study shows that our roller, the non‐occlusive roller, and centrifugal pump, all show suitable flow dynamics with adjustable flow range, although overall producing non‐pulsatile flow. Neither of the three pumps causes detectable disturbances to the MR imaging process, however, the electrical components were not yet tested. To further assess these three prototypes, a hemolysis study will be conducted, assessing the impact of the pump on the red blood cells before assessing the impact of a HLM on cerebral perfusion dynamics in vivo.

## Author Contributions

Dominik T. Schulte: concept, hardware development, conducting experiments, data analysis, writing of article. Marcel Renggli: hardware development, conducting experiments, data analysis. Henning Richter: conducting experiments, data analysis. Francesca Del Chicca: conducting experiments, data analysis. Michael Hofmann: hardware development, securing funding. Martin Schmiady: securing funding. Marianne Schmid Daners: concept, hardware development, securing funding, critical revision of article. All authors reviewed and approved the final version of the manuscript.

## Conflicts of Interest

The authors declare no conflicts of interest.

## Data Availability

The data that support the findings of this study are available from the corresponding author upon reasonable request.
